# Interferon Beta and Vitamin D Synergize to Induce Immunoregulatory Receptors on Peripheral Blood Monocytes of Multiple Sclerosis Patients

**DOI:** 10.1371/journal.pone.0115488

**Published:** 2014-12-31

**Authors:** Anne Waschbisch, Nicholas Sanderson, Markus Krumbholz, George Vlad, Diethilde Theil, Stefan Schwab, Mathias Mäurer, Tobias Derfuss

**Affiliations:** 1 Dept. of Neurology, Friedrich-Alexander University Erlangen-Nürnberg, Erlangen, Germany; 2 Dept. of Neurology and Biomedicine, University Hospital Basel, Basel, Switzerland; 3 Institute of Clinical Neuroimmunology, Klinikum Grosshadern, Ludwig Maximilian University, Munich, Germany; 4 Dept. of Pathology & Cell Biology, Columbia University, New York, New York, United States of America; 5 Dept. of Neurology, Klinikum Grosshadern, Ludwig Maximilian University, Munich, Germany; National Institutes of Health, United States of America

## Abstract

Immunoglobulin-like transcript (ILT) 3 and 4 are inhibitory receptors that modulate immune responses. Their expression has been reported to be affected by interferon, offering a possible mechanism by which this cytokine exerts its therapeutic effect in multiple sclerosis, a condition thought to involve excessive immune activity. To investigate this possibility, we measured expression of ILT3 and ILT4 on immune cells from multiple sclerosis patients, and in post-mortem brain tissue. We also studied the ability of interferon beta, alone or in combination with vitamin D, to induce upregulation of these receptors in vitro, and compared expression levels between interferon-treated and untreated multiple sclerosis patients. *In vitro* interferon beta treatment led to a robust upregulation of ILT3 and ILT4 on monocytes, and dihydroxyvitamin D3 increased expression of ILT3 but not ILT4. ILT3 was abundant in demyelinating lesions in postmortem brain, and expression on monocytes in the cerebrospinal fluid was higher than in peripheral blood, suggesting that the central nervous system milieu induces ILT3, or that ILT3 positive monocytes preferentially enter the brain. Our data are consistent with involvement of ILT3 and ILT4 in the modulation of immune responsiveness in multiple sclerosis by both interferon and vitamin D.

## Introduction

Immunoglobulin-like transcripts (ILT), also known as leukocyte immunoglobulin-like receptors (LILR) belong to a large family of activating and inhibitory receptors that modulate the threshold and amplitude of immune cell activation [1], [2]. Immune inhibitory ILT family members such as ILT3 (CD85k, LILRB4) and ILT4 (CD85d, LILRB2) are characterized by an extracellular immunoglobulin-like domain responsible for ligand-binding, and a long cytoplasmatic tail containing immunoreceptor-tyrosine based inhibitory motifs (ITIM) which recruit inhibitory phosphatases and transduce a negative signal into the cell [3]. A high expression of ILT3 and ILT4 on the cell surface of antigen-presenting cells renders them tolerogenic, inhibiting T cell proliferation and favouring the generation of CD8+ T suppressor cells [4], [5], [6], [7].

Multiple sclerosis (MS) is a chronic, inflammatory, demyelinating disease of the central nervous system (CNS). It has been hypothesized that an aberrant activation of autoreactive T cells in the peripheral immune compartment due to failure of peripheral tolerance mechanisms triggers T-cell mediated CNS inflammation in MS [8]. Accordingly, impaired expression of molecules involved in the regulation of T cell activation such as PD-1 and other B7 family members has been found to exacerbate CNS inflammation in animal models of the disease [9], [10], [11]. While the role of ILT3 and ILT4 as regulators of T cell activation and mediators of tolerance is well recognized in transplant and cancer immunology [12], [13], [14], little is known about the influence of these receptors on autoimmune diseases.

Here we show that the beneficial effects of IFN beta in multiple sclerosis may in part be mediated by modulation of ILT3 and ILT4 expression on APC.The role of immunoregulatory receptors in CNS inflammation is further highlighted by their enrichment in cerebrospinal fluid and an upregulation of ILT3, ILT4, and B7-H3 in acute MS lesions.

## Results

### ILT3 and ILT4 expression on monocytes is upregulated by in vitro IFN beta treatment

**Figure 1:**
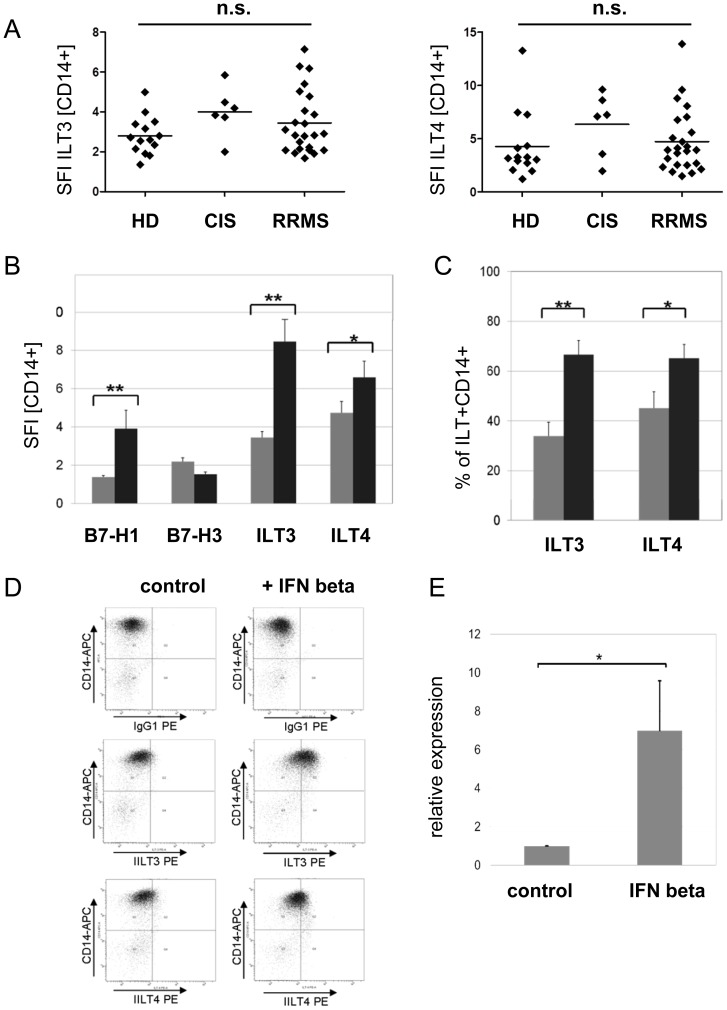
IFN beta induces ILT3 and ILT4 expression in monocytes. (A) The baseline expression of the immune inhibitory receptors ILT3 and ILT4 on peripheral blood derived CD14+ monocytes assessed by comparing the specific fluorescent index (SFI  =  geo mean ILT/geo mean IgG1) does not differ significantly between RRMS (n = 24) or CIS patients (n = 6) and healthy donors (HD, n = 14) as demonstrated by flow cytometry. (B) In vitro IFN beta (1000IE/ml, 16 h) treatment induces ILT3 and to a lesser extent ILT4 protein expression in CD14+ monocytes from RRMS patients (n = 24). In addition the monocytic expression of the coinhibitory B7 family member B7-H1 is upregulated, whereas B7-H3 is not significantly affected. The specific fluorescent index is shown (grey bar: control; black bar: IFN beta). (C) The percentage of CD14+ monocytes expressing the immune inhibitory ILT is strongly increased following incubation with IFN beta as assessed by flow cytometry (n = 24; grey bar: control; black bar: IFN beta 1000 IE/ml, 16 h). (D) A representative flow cytometry experiment is shown. Please note, for simplification the isotypic control for ILT4 (IgG2a) is not shown in this picture. (E) IFN beta induces ILT3 mRNA in purified CD14+ monocytes derived from healthy donors (n = 6). Relative expression of ILT3 mRNA was assessed by quantitative PCR (Mann-Whitney U test; * p<0,05, ** p<0,005; bars are presented as mean ± S.E.M)

CD14+ monocytes derived from treatment-naïve patients with RRMS (n = 24) or CIS (n = 6) and healthy controls (n = 14) were analyzed for surface expression of ILT3 and ILT4 by flow cytometry. Baseline expression of ILT3 and ILT4 as assessed by comparing the specific fluorescent indices (SFI) did not differ significantly between these groups (Fig. 1A). Regarding the percentage of ILT3 positive cells, CIS patients tended to have higher numbers of ILT3+CD14+ cells (mean +/-S.E.M.: 55.03+/−5.85; p<0.05) compared to RRMS patients (34.78 +/−5.73) and healthy controls (25.54 +/−4.16). No significant differences concerning the percentages of ILT4+CD14+ cells were observed between these groups (data not shown).

Furthermore, we could not detect any differences regarding the baseline expression of B7-H1 and B7-H3 (data not shown). *In vitro* IFN beta treatment of PBMC resulted in a strong upregulation of both ILT3 and ILT4 on CD14+ monocytes (Fig. 1 B, D). The percentage of ILT3+ monocytes was almost doubled from 35% to 68% and the percentage of ILT4 monocytes increased from 47% to 66% (Fig. 1C). The extent of ILT3 and ILT4 induction was comparable to that of B7-H1, a known target protein of IFN beta [15], whereas the expression of the novel B7-homologue B7-H3 remained unaffected (Fig. 1 B). Upregulation of ILT3 by IFN beta was induced in isolated monocytes indicating that lymphocytes do not contribute to this effect (Fig. 1 E). Upregulation of CD86 expression was monitored as a positive control in each experiment (data not shown).

### Additive effects of IFN beta and Vitamin D3 on monocytic ILT3 expression

**Figure 2:**
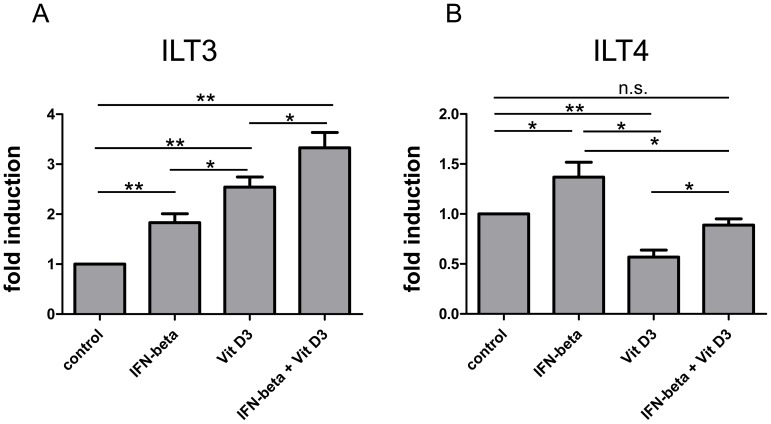
Effects of 1α,25 Dihydroxyvitamin D3 in combination with IFN beta on monocytic ILT3 and ILT4 expression. PBMC derived from RRMS patients (n = 9) were stimulated with 1α,25 Dihydroxyvitamin D3 (100 nM) and/or IFN beta (1000 IE) over a 48 h period. ILT3 and ILT4 protein expression on CD14+ cells was assessed by flow cytometry. The fold induction is shown (mean + SEM). A repeated measurement ANOVA with Bonferroni multiple comparison test was performed to assess statistical significance (* p<0,05; **p<0.005).

1α,25 Dihydroxyvitamin D3 (1α,25(OH)_2_D3) has been reported to induce ILT3 but not ILT4 expression on antigen-presenting cells [16]. Using flow cytometry we assessed the impact of 1α,25(OH)_2_D3 (100 nM) alone or in combination with IFN beta (1000 IE) on the expression of these immunoregulatory molecules on the surface of CD14+ monocytes derived from a second cohort of RRMS patients (n = 9, Fig. 2) and healthy controls (n = 8, S1 Fig.). In line with previous findings 1α,25(OH)_2_D3 was found to be a potent inducer of ILT3 but not ILT4 expression. Combined stimulation with IFN beta had a strong additive effect, leading to high levels of membrane bound ILT3 protein on CD14+ cells (Fig. 2). In contrast ILT4 levels, which were downregulated in 1α,25(OH)_2_D3 only treated cells, were restored back to normal levels in the presence of both reagents (Fig. 2). Similar trends were observed in healthy controls; however, the downregulation of ILT4 by 1α,25(OH)_2_D3 and the restoration of ILT4 surface levels by a combined treatment did not reach the level of significance in controls (S1 Fig.).

### ILT3 mRNA is upregulated in PBMC from IFN beta treated RRMS patients

**Figure 3:**
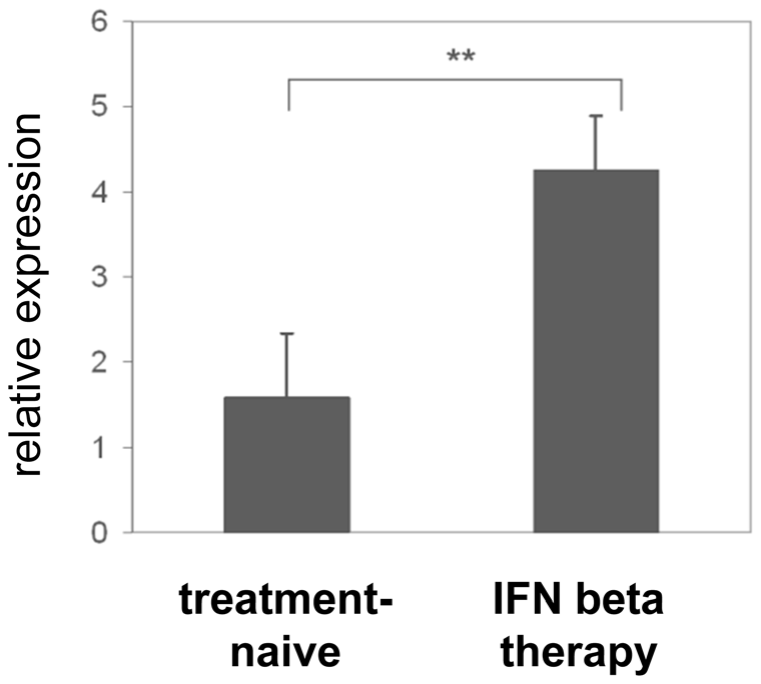
ILT3 mRNA is upregulated in PBMC derived from IFN beta treated RRMS patients. Relative expression of ILT3 mRNA in PBMC derived from IFN beta treated patients with RRMS (n = 10) and sex- and age matched controls (n = 10) as assessed by quantitative PCR (Mann-Whitney U test, **p<0,005). Bars are presented as mean ± S.E.M.

To corroborate our *in vitro* findings, we analyzed the expression of ILT3 mRNA in PBMC derived from treatment naïve RRMS patients and patients undergoing long-term treatment with either IFN beta-1b (Betaferon) or IFN beta-1a (Rebif) by quantitative PCR. In line with our *in vitro* findings, expression of ILT3 mRNA was 2.5 fold higher in IFN beta treated compared to untreated RRMS patients (Fig. 3). Of note, there were no differences between the two preparations of IFN beta concerning the expression of ILT3 mRNA.

### Soluble ILT3 serum concentrations do not seem to be affected by IFN beta treatment

In addition to membrane-bound ILT3, soluble ILT3 (sILT3) has been described and presumed to result from proteolytic cleavage from the cell surface or from alternatively spliced ILT3 isoforms that lack the transmembrane domain. Previously, sILT3 was detected in sera from patients with various malignancies, giving rise to the hypothesis that sILT3 may play a role in the immune evasion of tumor cells [12]. We asked whether patients with multiple sclerosis (n = 15) would show detectable serum levels of sILT3 and whether these levels would be modulated by IFN beta treatment. Serum from melanoma patients about to start (n = 3) or already undergoing IFN alpha therapy (n = 8) served as a control. We failed to detect sILT3 in all but 2 patients with RRMS which is in the range of healthy controls [12]. From one of the RRMS patients displaying detectable levels of sILT3 protein two samples from before and 3 months after initiation of IFN beta therapy were available. Interestingly these showed a remarkable consistency over time without any impact of IFN beta therapy (before: 26 ng/ml, after: 28 ng/ml; S1 Table). Also from 8 patients with malignant melanoma, only 2 had detectable levels of sILT3. An effect of IFN alpha treatment was not detected in the 3 melanoma patients in which pre- and post-treatment samples were available (S1 Table).

### ILT3 expressing monocytes in CSF

**Figure 4:**
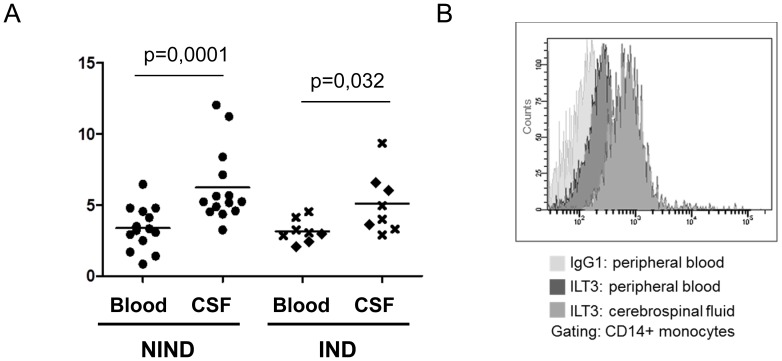
CSF monocytes express high levels of ILT3. (A) Paired blood and CSF samples were analyzed for expression of ILT3 on CD14+ monocytes by flow cytometry. The specific fluorescent index is shown. Patients were categorized according to their diagnosis as having non-inflammatory neurological diseases (NIND, n = 14; marked by dot) or inflammatory neurological disease (IND, n = 8) including 5 MS patients (marked by x) and 3 patients with other inflammatory neurological disease (diamond). A Mann-Whitney U test was performed to assess statistical significance. (B) Histogram of a representative staining in a patient with NIND.

Flow cytometry of paired blood and CSF specimens demonstrated a higher level of ILT3 surface protein on CSF-derived than on blood-derived CD14+ monocytes, both in patients with and without neuroinflammatory diseases (Fig. 4A, B). Accordingly the percentage of ILT3+ CD14+ monocytes in the CSF ranged from 45% up to 95% demonstrating an enrichment of ILT3+ monocytes in the CSF. There were no statistically significant differences between patients with non-inflammatory neurological diseases (n = 14) and those with inflammatory neurological diseases (n = 8) concerning the percentage of ILT3+ monocytes or the level of ILT3 expression. This holds true for both, CSF and blood samples in this cohort (Fig. 4A).

### Expression of the immunoregulatory molecules ILT3, ILT4, and B7-H3 in MS lesions and control tissue

**Figure 5:**
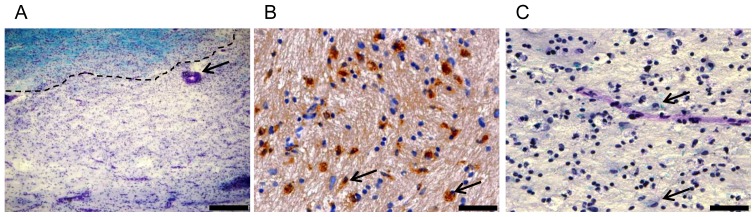
Characterization of MS tissue blocks. (A) Overview of a demyelinating lesion with perivascular cuffing. Myelin is stained blue with LFB. Lesion border is indicated by a dotted line. Perivascular cuff is marked by an arrow. Scale bar indicates 400 µm. (B) CD68 positive macrophages in demyelinated lesion. CD68 positive cells are stained brown and marked by arrows. Scale bar indicates 50 µm. (C) Demyelinated lesion in higher magnification with cells reminiscent of macrophages that phagocytosed LFB positive myelin debris (marked with arrows). Scale bar indicates 50 µm.

**Figure 6:**
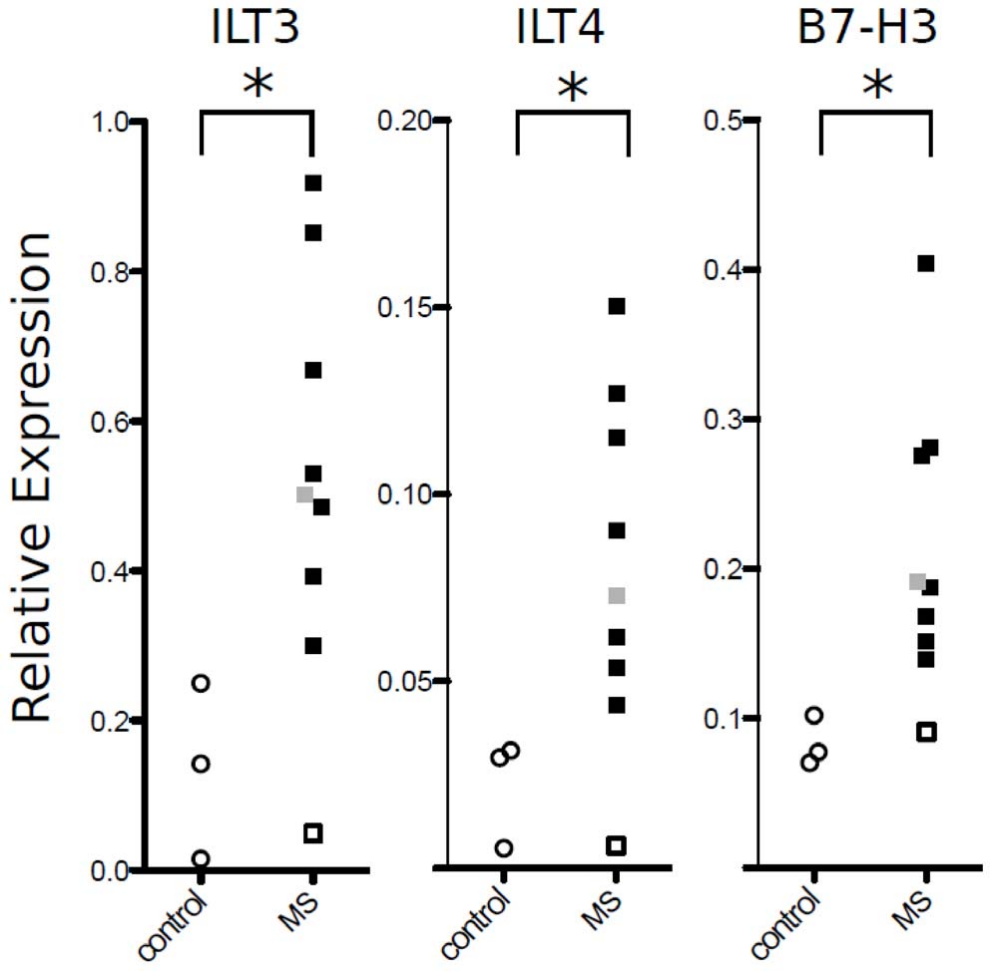
ILT3, ILT4 and B7-H3 mRNA expression in MS tissue samples. Expression of ILT3, ILT4, and B7-H3 in CNS autopsy samples measured by quantitative PCR and normalized to PUM1. For each gene, results from normal white matter from control brains are shown on the left (open circles), and results from 7 active lesions (black squares) and 1 inactive lesion (grey square) and 1 normal appearing white matter (open square) are shown on the right. Vertical axis shows the expression relative to PUM1 calculated according to the formula in the Methods section (*p<0.05, Mann-Whitney U test).

Lesions were characterized and graded for the activity of demyelination by luxol fast blue, hematoxylin/eosin staining and CD68 immunohistochemistry (Fig. 5). Lesions were characterized as active when they showed recent demyelination, macrophage infiltration, and perivascular cuffing. Seven lesions were classified as active lesions. One lesion was defined as chronic inactive. As controls, three samples from white matter of control brains and one sample from normal appearing white matter of a MS brain were included. Quantitative RT-PCR revealed an upregulation of ILT3, ILT4, and B7-H3 transcripts in active lesions and in the one chronic inactive lesion compared to control brain and normal appearing white matter of a MS brain (Fig. 6). B7-H1 has been previously reported to be upregulated on microglial/macrophage-like cells within active MS lesions. Due to the limited amount of human material, we here refrained from reproducing the results on B7-H1 obtained by Ortler et al. [17].

## Discussion

The immune system has to preserve immune tolerance to self while maintaining the ability to fight foreign pathogens. In addition to central tolerance mechanisms, the control of T cell activation in the periphery is essential to prevent autoimmunity [18]. Both antigen-presenting cells and T cells are endowed with a large repertoire of activating and inhibitory molecules. It is the integration of these signals that keeps T cells in check and dictates the outcome of an APC-T cell encounter [11], [19]. Here we report that IFN beta, an immunomodulatory drug widely used to treat multiple sclerosis, induces the expression of the immune inhibitory receptors ILT3 and ILT4 on monocytes. Our findings confirm *in vitro* data by Jensen et al. [20] and further show that upregulation of monocytic ILT3 expression by IFN beta is not an *in vitro* artefact, but can also be found in patients with relapsing-remitting multiple sclerosis undergoing IFN beta therapy as demonstrated by quantitative PCR. In addition, we demonstrate that the extent of ILT3 and ILT4 upregulation in monocytes is comparable to that of the coinhibitory molecule B7-H1 which has been reported as a target of IFN beta therapy in MS [21]. In contrast, the expression level of the coinhibitory B7 homologue B7-H3 remained unaffected, pointing to a selective effect of IFN beta on these immunomodulatory molecules and monocyte immunobiology.

Vitamin D insufficiency has emerged as a potential risk factor for multiple sclerosis and may be associated with a higher disease activity [22], [23], [24], [25]. Larger studies addressing effects of oral high-dose vitamin D are on their way [26], including a phase II trial of vitamin D and IFN beta cotreatment (SOLAR, NCT01285401). A previous clinical study suggested that vitamin D and IFN beta act together in modulating disease activity in MS [27]. However, on a cellular level the mechanisms of this interaction remain elusive. The bioactive form of vitamin D, 1α,25(OH)_2_D3 has previously been reported to be a potent inducer of ILT3 on APC [16]. Here we demonstrate that IFN beta and 1α,25(OH)_2_D3 work together in inducing ILT3 receptor expression in monocytes. Thereby, vitamin D co-treatment could contribute to the beneficial effects of the disease-modifying drug. Interestingly, IFN beta and 1α,25(OH)_2_D3 have contrasting effects on ILT4 expression levels on monocytes. By counteracting the effects of 1α,25(OH)_2_D3 on ILT4 expression IFN beta may further contribute to the tolerogenic properties of these cells.

Interestingly, ILT3 has bidirectional signalling properties [28]. In addition to the signal transduced via its intracellular domain, the extracellular Ig-like domain of ILT3 binds to T cells and may directly modulate. [28]. Accordingly, soluble forms of ILT3 are effective inhibitors of T cell proliferation, even in the absence of APC and may be of interest for future therapeutic use as immunomodulators in autoimmune diseases. [6], [12], [28], [29].

So far, ILT3 serum levels have not yet been systematically analyzed in patients with autoimmune diseases. In our study only 2 out of 15 MS patients tested positive for soluble ILT3 in serum, which is in the range of healthy individuals [12]. As a positive control we studied patients with melanoma. In contrast to a previous publication [12], the rate of detectable sILT3 in serum was low in this cohort (∼25%). The discrepancy of our findings may be due to the fact that the previous publication was confined to patients with advanced-stage melanoma, while our cohort also included patients that, although at high risk for spreading, still had localized disease. In addition the patients examined by Suciu-Foca et al were enrolled for treatment with high-dose IL-2 which may also account for higher sILT3 serum levels in their cohort [12].

Current concepts of MS pathogenesis presume that breakdown of immune tolerance in the periphery is an initiating step in the cascade that finally leads to CNS infiltration by autoreactive T cells, demyelination and axonal loss [11]. Given the potent inhibitory capacity of ILT3 on T cell activation and proliferation, upregulation of this immune inhibitory receptor on monocytes in IFN beta-treated MS patients may indeed represent a therapeutic mechanism that helps to prevent overshooting immune responses. Demonstration of increased ILT3 expression on CSF monocytes and within active MS lesions suggests that the immunomodulatory effects of ILT3 are not restricted to the peripheral immune compartment but may also play a role at the site of inflammation in MS. Compared to the peripheral blood, CSF monocytes display high levels of ILT3 on their surface, with ILT3+ monocytes accounting for approximately two thirds of CD14+ cells in the CSF. This enrichment of ILT3+ monocytes in CSF can be observed not only in patients with inflammatory but also in individuals with non-inflammatory neurological diseases. Accordingly, the high expression of ILT3 on CSF monocytes seems not to be reactive to neuroinflammation but rather a primary attribute of monocytes in the CSF, suggesting a possible involvement of ILT3 in the basic mechanisms of CNS immune surveillance. However, since CSF was not available from healthy donors this remains speculative.

Silencing of ILT3 in monocyte-derived dendritic cells potentiates stimulus-induced release of chemokines, that may be involved in T cell trafficking to the CNS [5]. Therefore, ILT3 expressing monocytes within CSF-filled spaces have the potential not only to dampen T cell activity but also to counteract the attraction of pathogenic cells. Further evidence for a possible role of ILT3 at the site of inflammation in MS comes from our expression analysis demonstrating the presence of ILT3 in active MS lesions but not in normal-appearing white matter and white matter from control brains. Since immunohistochemical staining of ILT3 in brain tissue could not be established with the available antibodies we cannot tell the exact cellular source of ILT3 expression. However, since ILT3 expression is confined to professional and non-professional APC [2], [29] it can be assumed that macrophages and microglia account for a large fraction of the ILT3-expressing cells. Similar to ILT3, ILT4 was also found to be upregulated in active MS lesions.However, in contrast to ILT3, ILT4 was not analyzed on CSF monocytes in this study which limits the conclusions drawn from this finding.

This work provides *in vitro* and *ex vivo* evidence for an induction of the immune inhibitory molecules ILT3 and ILT4 on APCs by IFN beta. The IFN mediated induction of ILT3 can also be potentiated by vitamin D. Increased expression of ILT3 on CSF monocytes and in MS lesions together with other inhibitory receptors indicate a pivotal role of these molecules in the regulation of neuroinflammation. Targeting ILT3 may therefore represent a future therapeutic option in autoimmune diseases.

## Materials and Methods

### Clinical Samples

The study was approved by the local ethics committee (Nr. 4203, University Erlangen-Nuremburg). Written informed consent was obtained from all patients and and all clinical investigations have been conducted according to the principles expressed in the Declaration of Helsinki. Patients with relapsing-remitting multiple sclerosis (RRMS) according to the revised McDonald criteria (2005) [30] and clinically isolated syndrome (CIS) were eligible for participation in this study. All patients were naïve to immunomodulatory (IFN beta, glatiramer acetate, dimethylfumarate, teriflunomide, fingolimod, natalizumab, alemtuzumab) and immunosuppressive drugs (mitoxantrone, cyclophosphamide) except for patients presented in Fig. 3 from whom samples were obtained before and at least 3 months after initiation of IFN beta treatment and patients whose serum was obtained for sILT3 analysis. The individual treatment status with IFN beta in serum donors is stated in S1 Table. Patients treated with intravenous glucocorticoids or plasmapheresis during the preceding six weeks were excluded. In addition age- and sex-matched healthy volunteers were recruited. Control sera were obtained from patients with malignant melanoma treated at the Ludwig-Maximilian University of Munich and the University of Regensburg. Again the individual treatment status with IFN is indicated in S1 Table.

CSF and paired blood samples were collected from patients that were referred to the Dept. of Neurology of the University of Erlangen for diagnostic lumbar puncture. Patients were categorized into two groups: inflammatory neurological diseases (IND, e.g. viral encephalitis, multiple sclerosis) and non-inflammatory neurological diseases (NIND, e.g. normal pressure hydrocephalus). None of the CSF donors were undergoing IFN beta treatment.

Nine MS and three control tissue samples were supplied by the UK Multiple Sclerosis Tissue Bank (UK Multicentre Research Ethics Committee, MREC/02/2/39), funded by the Multiple Sclerosis Society of Great Britain and Northern Ireland (registered charity 207495). Autoptic brain samples were analysed by luxol fast blue, hematoxylin/eosin staining and CD68 immunohistochemistry as previously described [31]. Information on the pre-mortem treatment of these patients with disease modifying drugs was not available.

### Isolation and cultivation of peripheral blood mononuclear cells

PBMC were isolated by centrifugation on a Lymphoprep (Fresenius Kabi Norge AS, Oslo, Norway) density gradient and cultured in RPMI 1640 (Gibco Invitrogen GmbH, Karlsruhe, Germany) supplemented with 10% of heat inactivated human AB serum (PAA, Pasching, Austria), glutamine and antibiotics. Cells were grown at a densitiy of 2×10^6^/ml in 12-well plates (Gibco Invitrogen GmbH, Karlsruhe, Germany) under standard conditions (37°C, 5% CO_2_). For some experiments monocytes were purified from PBMC using anti-CD14 microbeads (Miltenyi Biotech GmbH, Bergisch Gladbach, Germany) and magnetic cell separation (MACS technology). IFN beta-1a (Rebif, 1000 IE/ml) or 1α,25-Dihydroxyvitamin D3 (100 nM) were added to the medium as indicated.

### Flow cytometry

PBMC were washed twice followed by Fc receptor blocking with human IgG (Sigma-Aldrich, Munich, Germany). Afterwards cells were incubated for 30 minutes at 4°C with specific monoclonal antibodies: anti-CD14 APC (MOP9, BD Bioscience, Heidelberg, Germany), anti-CD86 PE (IT2.2, eBioscience, Frankfurt, Germany), anti-B7-H1 PE (MIH1, eBioscience, Frankfurt, Germany), anti-B7-H3-PE (RnD Systems), anti-ILT3 PE (ZM4.1, eBioscience, Frankfurt, Germany), anti-ILT4 PE (42D1, eBioscience, Frankfurt, Germany) and the respective fluorochrome-conjugated isotypic controls.

For CSF analysis, cells were pelleted by centrifugation and resuspended in 100 µl of 4°C PBS 1% BSA within 30 minutes after the lumbar puncture. CSF and paired EDTA blood samples were stained with APC conjugated anti-CD14 antibody and PE-conjugated anti-ILT3 antibody or the respective isotype controls (blood only). Following erythrocyte lysis, cells were analyzed on a FACSCanto II (BD Biosciences, Heidelberg, Germany). Percentages of positive cells, as well as the specific fluorescent indices [geometric mean of the specific antibody fluorescence divided by the geometric mean of the isotype control antibody fluorescence] were calculated.

### RNA isolation and quantitative PCR

RNA from PBMCs was isolated using the Rneasy Mini Kit (Qiagen, Hilden, Germany). RNA from frozen tissue sections was purified using RNAzol (Sigma-Aldrich, Taufkirchen, Germany) according to the manufacturer's instructions, but with two subsequent phase separation steps to improve RNA purity. RNA was transcribed using random hexamers and MuLV reverse transcriptase (Promega, Mannheim, Germany) or high capacity cDNA Kit (ABI, Darmstadt, Germany). For expression analysis in PBMCs, PCR and quantification of PCR products was performed using Ssofast EVA Green Supermix (Biorad Laboratories, Hercules, CA, USA) on a MyIQ5 Real Time PCR System. The following primers for ILT3 were used during the amplification: forward, 5′-CTGCCGAGTCCTCTTGTGACC-3′; reverse, 5′ TGG AGG ACG TTG GAA ATC AGC-3′ [12]. Beta-Actin was used as an endogenous control (forward, 5′-AGG ATG CAG AAG GAG ATC ACT-3′, reverse 5′-GGG TGT AAC GCA ACT AAG TCA TAG-3′). Quantification of gene expression was performed according to the comparative cycle threshold method (2^−ΔΔCT^). Samples were normalized to beta-actin and compared to a reference sample used in all the experiments. Expression of ILT3, ILT4, B7-H3 and PUM1 in autopsy samples was measured using an Applied Biosystems 7500 with probes supplied by Applied Biosystems (Taqman Gene Expression Assays Hs00429000, Hs01629548, Hs00987207, and Hs00206469, respectively). Relative expression was calculated using PUM1 levels for normalisation.

### Sandwich ELISA detection of soluble ILT3

Soluble ILT3 ELISA was performed as previously described [12]. In brief, Maxisorp 96-well plates (Nalge Nunc International) were coated overnight with 1.0 µg/well anti-ILT3 mAb (clone ZL5.7). Free binding sites were blocked with 5% BSA solution for 1 h at room temperature. After washing with PBS with 0.1% Tween 20, serum samples were added at a 1∶3 and 1∶9 dilution and incubated for 1 hour. Detection was performed using 100 µl of biotinylated polyclonal anti-ILT3 antibody (R&D Systems) at a concentration of 33 ng/ml followed by HRP-conjugated streptavidin (BD Biosciences). Tetramethylbenzidine was used as a substrate.

## Supporting Information

S1 Fig **Effects of 1α,25 Dihydroxyvitamin D3 in combination with IFN beta on ILT3 and ILT4 expression in healthy donors.** PBMC derived from RRMS patients were stimulated with 1α,25 Dihydroxyvitamin D3 (100 nM) and/or IFN beta (1000IE) over a 48 h period. ILT3 and ILT4 protein expression on CD14+ cells was assessed by flow cytometry. The fold induction is shown (mean + SEM). A repeated measurement ANOVA and Bonferroni multiple comparison test was performed to assess statistical significance (* p<0.05; **p<0.005).
(TIF)

S1 Table **Soluble ILT3 in serum samples derived from MS or malignant melanoma patients.** Serum samples were analyzed for *soluble* ILT3 protein content by ELISA. From some of the RRMS and melanoma patients, samples derived from before (untreated) and after initiation of IFN beta or IFN alpha therapy (IFN therapy) were available. A slash (/) indicates a missing sample, n.d. (not detectable) indicates that soluble ILT3 serum concentrations were below the lower level of detection of the assay. Detectable levels of ILT3 were found in 2 patients with RRMS and 2 patients with melanoma. In contrast to the increased *surface* ILT3 expression after IFN beta treatment, *soluble* ILT3 concentrations were not elevated (at least not above the detection threshold) by IFN beta therapy.
(TIF)
