# Supplementary figures and images for: Interferon Beta and Vitamin D Synergize to Induce Immunoregulatory Receptors on Peripheral Blood Monocytes of Multiple Sclerosis Patients
Source: PLoS One. 2014 Dec 31;9(12):e115488. doi: 10.1371/journal.pone.0115488 (PMC4281069; doi:10.1371/journal.pone.0115488)

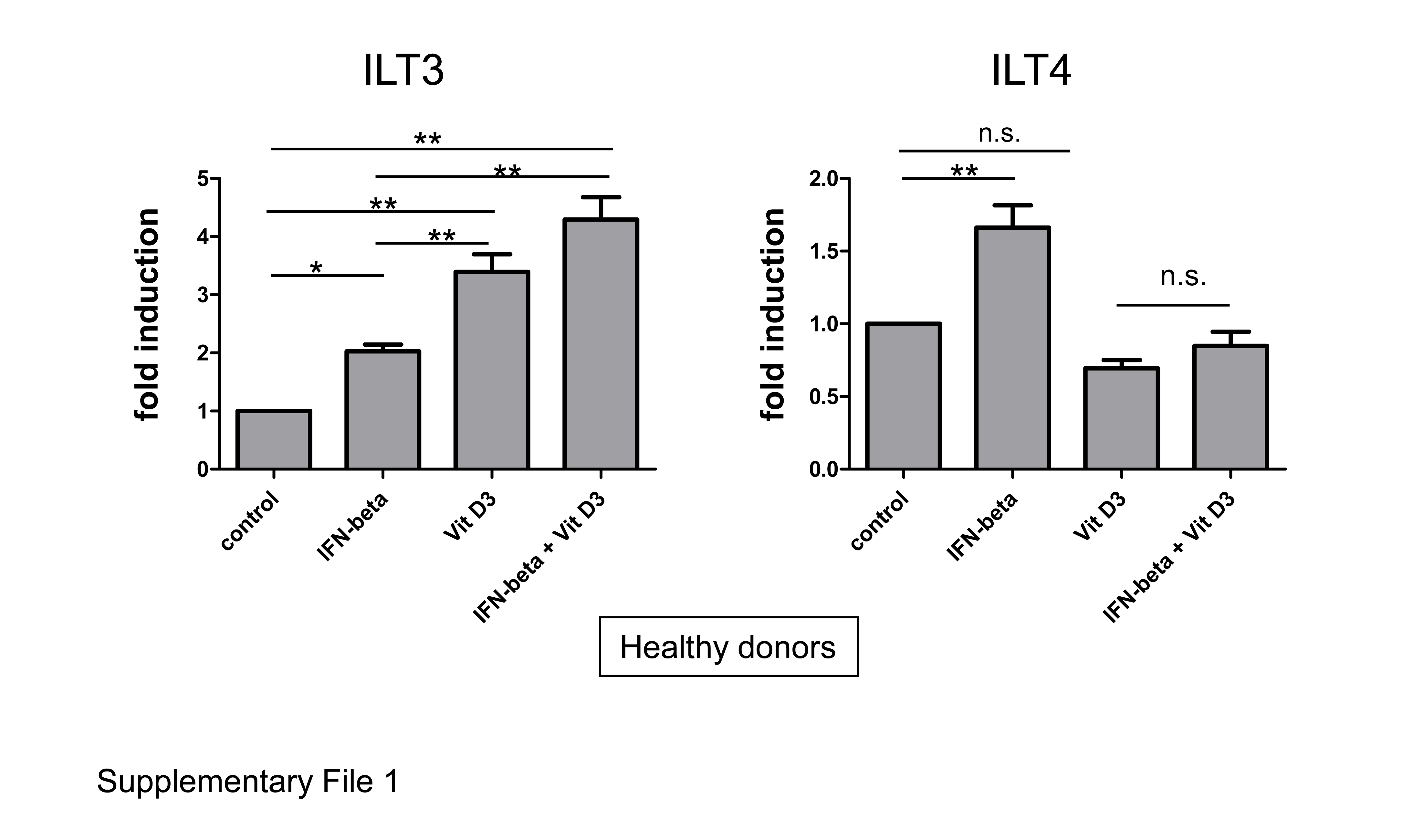

Supplement: S1 Fig — Effects of 1α,25 Dihydroxyvitamin D3 in combination with IFN beta on ILT3 and ILT4 expression in healthy donors. PBMC derived from RRMS patients were stimulated with 1α,25 Dihydroxyvitamin D3 (100 nM) and/or IFN beta (1000IE) over a 48 h period. ILT3 and ILT4 protein expression on CD14+ cells was assessed by flow cytometry. The fold induction is shown (mean + SEM). A repeated measurement ANOVA and Bonferroni multiple comparison test was performed to assess statistical significance (* p<0.05; **p<0.005). (TIF) [file pone.0115488.s001.tif]

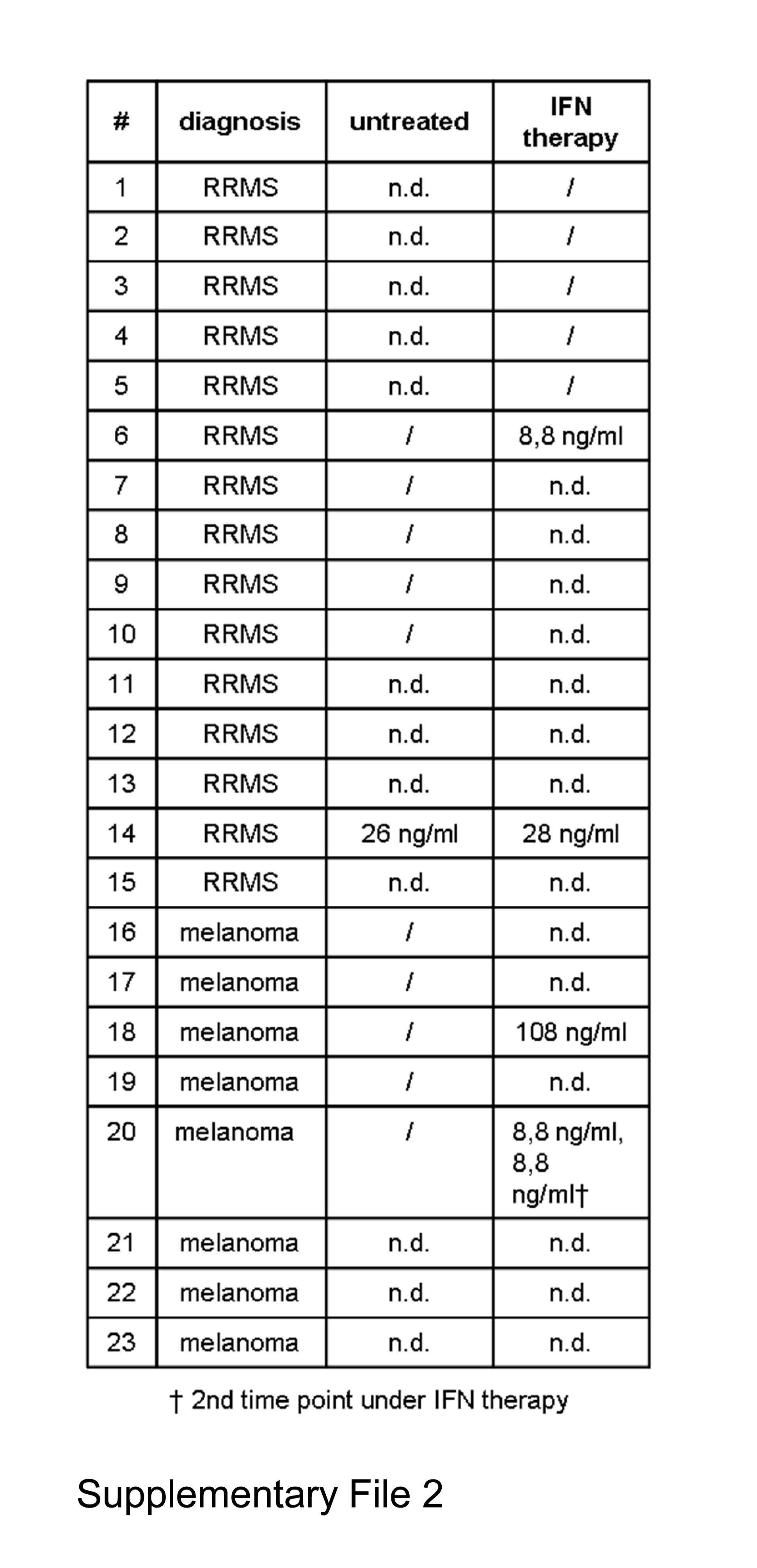

Supplement: S1 Table — Soluble ILT3 in serum samples derived from MS or malignant melanoma patients. Serum samples were analyzed for soluble ILT3 protein content by ELISA. From some of the RRMS and melanoma patients, samples derived from before (untreated) and after initiation of IFN beta or IFN alpha therapy (IFN therapy) were available. A slash (/) indicates a missing sample, n.d. (not detectable) indicates that soluble ILT3 serum concentrations were below the lower level of detection of the assay. Detectable levels of ILT3 were found in 2 patients with RRMS and 2 patients with melanoma. In contrast to the increased surface ILT3 expression after IFN beta treatment, soluble ILT3 concentrations were not elevated (at least not above the detection threshold) by IFN beta therapy. (TIF) [file pone.0115488.s002.tif]
